# Potential Utility of Retinal Imaging for Alzheimer’s Disease: A Review

**DOI:** 10.3389/fnagi.2018.00188

**Published:** 2018-06-22

**Authors:** Huan Liao, Zhuoting Zhu, Ying Peng

**Affiliations:** ^1^Department of Neurology, Sun Yat-sen Memorial Hospital, Sun Yat-sen University, Guangzhou, China; ^2^Guangdong Provincial Key Laboratory of Malignant Tumor Epigenetics and Gene Regulation, Sun Yat-sen Memorial Hospital, Sun Yat-sen University, Guangzhou, China; ^3^State Key Laboratory of Ophthalmology, Zhongshan Ophthalmic Center, Sun Yat-sen University, Guangzhou, China

**Keywords:** Alzheimer’s disease, retinal imaging, early detection, novel biomarker, review

## Abstract

The ensuing upward shift in demographic distribution due to the increase in life expectancy has resulted in a rising prevalence of Alzheimer’s disease (AD). The heavy public burden of AD, along with the urgent to prevent and treat the disease before the irreversible damage to the brain, calls for a sensitive and specific screening technology to identify high-risk individuals before cognitive symptoms arise. Even though current modalities, such as positron emission tomography (PET) and cerebrospinal fluid (CSF) biomarker, showed their potential clinical uses in early detection of AD, the high cost, narrow isotope availability of PET probes and invasive characteristics of CSF biomarker limited their broad utility. Therefore, additional tools for detection of AD are needed. As a projection of the central nervous system (CNS), the retina has been described as a “window to the brain” and a novel marker for AD. Low cost, easy accessibility and non-invasive features make retina tests suitable for large-scale population screening and investigations of preclinical AD. Furthermore, a number of novel approaches in retina imaging, such as optical coherence tomography (OCT), have been developed and made it possible to visualize changes in the retina at a very fine resolution. In this review, we outline the background for AD to accelerate the adoption of retina imaging for the diagnosis and management of AD in clinical practice. Then, we focus on recent findings on the application of retina imaging to investigate AD and provide suggestions for future research directions.

## Introduction

Life expectancy has increased substantially during past few decades, mainly attributable to advancements of health care and lifestyle. The ensuing upward shift in demographic distribution has resulted in a rising prevalence of aging-related diseases, such as dementia. According to the report in Prince et al. (2015), dementia affects approximately 46.8 million people worldwide. AD, as the most prevalent type of senile dementia by far, accounts for 60–80% of all cases with dementia (Prince et al., 2015). The prevalence of AD is estimated to quadruple and intensive health care will be needed for 43% of these patients by 2050 (Brookmeyer et al., 2007).

Despite of the heavy public disease burden of AD, there is no effective treatment for AD. Advances in the effective treatment and prevention of AD have been hampered by challenges in diagnosing the disease at the preclinical phase, in which subjects are still asymptomatic in clinical settings but may have subtle evidence of early cognitive deficits in the context of neuropathology specific to AD (Sperling et al., 2011a). Currently, diagnosis was primarily based on cognitive assessments among patients with symptomatic cognitive and behavior deficits (Dubois et al., 2014). Studies suggested that measurable changes in PET, MRI and CSF biomarkers occurred predates the onset of clinical symptoms (Beckett et al., 2010). Unfortunately, these are costly and/or invasive procedures that are not appropriate for screening at a population level.

As a projection of the CNS via the optic nerve, the retina has been described as a “window to the brain” and investigated intensively the potential of serving as a marker for AD (Guo et al., 2010; MacCormick et al., 2015). Low cost, easy accessibility and non-invasive features make retina tests suitable for large-scale population screening and investigations of preclinical AD. Furthermore, a number of novel approaches in retina imaging, such as OCT, have been developed and made it possible to visualize lesions and changes of the retina at a very fine resolution.

In this review, we outline the background for AD to accelerate the adoption of retina imaging for the diagnosis and management of AD in clinical practice. Then, we focus on recent findings on the application of retina imaging to investigate AD and provide suggestions for future research directions.

## Challenges for Early Detection of AD

In 1906, Alois Alzheimer was credited with identifying the first condition of dementia, which was later to carry his name as AD. AD is a progressive neurodegenerative disease, in which synaptic and neuronal loss(Terry et al., 1987; Polanco et al., 2018) lead to irreversible deterioration in memory loss, cognitive impairment and behavioral deficits(Ghiso et al., 2013). Well-known pathological hallmark related to AD is the propensity of proteins to form toxic oligomers and fibrils (Jahn et al., 2010). The two key proteins related to AD are Aβ peptide and hyper-phosphorylated tau (Masters et al., 1985; Grundke-Iqbal et al., 1986). Aβ peptide is a small peptide deprived from its parent molecule, the amyloid-β precursor protein (AβPP), and accumulates of Aβ into extracellular amyloid senile plaques (Polanco et al., 2018). Hyper-phosphorylated tau is a MAP that accumulates intraneuronally to form neurofibrillary tangles (Moore et al., 2016). Consistent evidences have demonstrated that neuropathologial changes, mainly the accumulation of Aβ and tau, predate the emergence of clinical symptoms by as long as 15–20 years, emphasizing the potential of developing methods for early diagnosis (Holtzman et al., 2011).

According to the National Institute of Neurological and Communicative Disorders and Stroke and the Alzheimer’s Disease and Related Disorders Association criteria (McKhann et al., 1984), the first definition for diagnosis of AD is relied on neuropathological analysis of brain tissue, obtained by biopsy or autopsy, for the accumulation of Aβ peptides and tau protein. In the following decades, advances in the early diagnosis of AD have been hampered by challenges in differentiating earliest stage of AD with age-related cognitive deficit and other forms of dementia. Furthermore, AD is currently diagnosed by cognitive assessments among patients with symptomatic cognitive and behavior deficits (Dubois et al., 2014), which is usually the advanced stage and irreversible to treatment. Despite of enormous financial and scientific efforts, the treatment of AD has remained symptom-driven and no single disease-modifying therapy for AD has been approved. So far, it has been documented the high failure rate – nearly 90% – of clinical drug trials for AD. One of possible reasons for the high failure rate might be the inclusion of patients with the uncertainty diagnosis and/or late-stage disease (Sperling et al., 2011b; James et al., 2015; Jellinger and Attems, 2015). Two phase 3 clinical trials(Doody et al., 2014; Salloway et al., 2014) investigated the anti-Aβ antibody’s effect on the treatment of mild-to-moderate AD. Neither study showed a significant benefit of solanezumab (Doody et al., 2014) or bapineuzumab (Salloway et al., 2014) for primarily designated outcomes. However, with sub-analysis of participants with mild AD, half of the prespecified endpoints was met and cognitive deficits were slowed by 34% (Siemers et al., 2016), suggesting the therapy may be effective in treating the early course of AD. A recent phase 1b randomized trial of monthly intravenous infusion of the anti-Aβ antibody abucanumab in patients with prodromal or mild AD did not have enough power to detect clinical change, however, *post hoc* analysis suggested the reduction of Aβ burden in brain and a stabilization of cognitive decline based on Clinical Dementia Rating-Sum of Boxes and Mini Mental State Examination scores (Sevigny et al., 2016). Modeling has suggested that interventions aimed at clearing Aβ accumulation may be sufficient to delay the onset of clinically manifest AD, reduce the incidence and the progression of AD if started in the early stages of AD (Brookmeyer et al., 1998). These findings again stress the importance of early detection of AD.

The increasing global prevalence of AD, along with the urgent to prevent and treat the disease before the irreversible damage to the brain, calls for a sensitive and specific screening strategy to identify individuals at high risk of AD before cognitive symptoms arise. A large number of novel methods for diagnosis of AD have been developed during the past decades. Non-invasive neuroimaging tools such as MRI can detect subclinical structural brain changes (e.g., atrophy) related to the future risk of AD. However, due to limited spatial resolution, MRI measures may not be able to detect subtle changes at the early stages. Although specific neuropathology biomarkers, such as the detection of Aβ and tau accumulation measured by PET imaging (James et al., 2015; Dubois et al., 2016) and in CSF (Bateman et al., 2012; Fagan et al., 2014) can be detected predates the onset of clinical symptoms and have shown high specificity in confirming AD pathophysiology (Beckett et al., 2010; Dubois et al., 2014), the high cost, narrow isotope availability of PET probes and invasive characteristics of CSF biomarkers limited their broad usages.

Therefore, additional tools for detection of AD are needed, with the aim of changing from an exclusionary approach to an accuracy diagnosis, from late-stage symptomatic diagnosis to the subclinical pathology detection, and using in large-scale population screening to identify individuals at high risk of developing AD. As the projection of CNS, the retina has attracted researchers’ attention to study AD and its pathology. A large number of studies have employed retina imaging for early detection and large-scale population screening of AD, and considerable progresses have been achieved in recent years.

### The Retina – A “Window to AD”

As an extension of the CNS, the retina and optic nerve share many features in terms of embryological origin, anatomy, response to injury, immunology and physiological characteristics, with the brain (London et al., 2013). The retina and optic nerve originate from the diencephalon during embryonic development, and therefore are considered as an extension of the CNS. Photoreceptor cells of the retina capture light and initiate neuronal signals that eventually reach the RGCs. The optic nerve composed of the axons of RGCs passes the visual information to the higher visual processing centers in the brain. RGCs show the typical patterns of CNS neurons and comprise a cell body, dendrites and an axon. Similar to all fiber tracts of CNS, the optic nerve is myelinated as they leave the eyes. Insult to the optic nerve leads to degeneration of the axons, myelin damage and inducing a neurotoxic condition, which are also observed in other CNS axons (Faden and Salzman, 1992; Schwartz et al., 1996; Crowe et al., 1997; Yoles and Schwartz, 1998; Levkovitch-Verbin et al., 2001, 2003). After injury, similar limited regeneration environment exists in the retina, optic nerve and other CNS compartments. The eye and the brain normally maintain strict interactions with immune system, and both are immune-privileged organs. Furthermore, ocular inner BRB resembles the BBB strongly with respect to structures, characteristics and mechanisms (Kaur et al., 2008).

## Retinal Changes and AD

Given these strong connections between the brain and the retina, it has been indicated that neurodegenerative disease, such as AD, may also lead to similar pathology in RGCs and optic nerve. The retina and brain are directly connected by axons of the optic nerve, which facilitate transportation of APP synthesized in RGCs in small transport vesicles (Morin et al., 1993). Furthermore, retinal neurons and glia express proteins that have participated in the amyloid cascade (e.g., BACE1, γ-secretase, APOE) (Cai et al., 2012; Li et al., 2016; Vecino et al., 2016). Several studies verified the presence of classical biomarkers of AD, Aβ and tau, within the retina and optic nerve at the molecular level (Gasparini et al., 2011; Koronyo-Hamaoui et al., 2011). Retinal Aβ deposits triggers breakdown of RPE tight junction (Park et al., 2014) and integrity of the blood-retina barrier (Dinet et al., 2012), increases reactive oxygen species production (Bruban et al., 2009), and activates complement by upregulating factor B (Wang et al., 2009). The crucial roles of Tau played in retina include regulating the cytoskeletal and axonal transport in retinal neurons, increasing Aβ accumulation and affecting cell survival signaling in the retina (Ho et al., 2012). Therefore, the toxic effects of these deposits include the apoptosis of the RGCs, thinning of the RNFL, morphological changes in the optic nerve, and other retinal structural and functional impairment of AD patients (Hinton et al., 1986; Tsai et al., 1991; Hedges et al., 1996; Danesh-Meyer et al., 2006; Iseri et al., 2006; Paquet et al., 2007; Ning et al., 2008; Perez et al., 2009; Parnell et al., 2012; Garcia-Martin et al., 2014; Salobrar-Garcia et al., 2015). Studies using animal models of AD have demonstrated that AβPP and Aβ were accumulated in all six layers of the neuroretina (Liu et al., 2009), with the extent proportional to the increasing age of the animal models. Furthermore, animal models have shown that retinal senile plaques were correlated with plaque load in the brain (Koronyo et al., 2012) and were detected prior to the plaque deposition in the brain, implying that Aβ deposition seen in the retina could be an early related sign of AD detection (Zhang-Nunes et al., 2006; Koronyo-Hamaoui et al., 2011). In addition, hyperphosphorlylated tau has been reported to accumulate in the RNFL of transgenic AD mouse models, which results in disruption of axonal transport and ganglion cell degeneration (Gasparini et al., 2011; Bull et al., 2012).

It has been suggested that AD might also be a disease of the neurovascular unit indicated from results of cerebral amyloid angiopathy, focal lesions in brain microcirculation system and strong associations between vascular changes of AD with typical neuronal degenerative changes (Zlokovic, 2011). The overproduction of Aβ in the retinal vascular increases the mechanical stress to endothelial cell regulation of AβPP, with associated destruction of the vessel walls, leading to changes of vascular diameters and topology (Golzan et al., 2017). A large body of evidences have also verified potential associations between AD and retinal vascular parameters, both static and dynamic, such as retinal vascular diameters, retinal pulsatility and local arterial PWV (Berisha et al., 2007; Pase et al., 2012; Frost et al., 2013; Feke et al., 2015; Williams et al., 2015). Transgenic animal models of AD have shown Aβ and senile plaques accumulation in retinal vasculature (Zhang-Nunes et al., 2006; Liu et al., 2009).

## Imaging Retina to Study AD

The visualization of detailed structure and functions of the retina has dramatically improved with the development of modern imaging techniques. This has provided clinicians and researchers with valuable tools for potential early diagnosis and differentiation, monitoring progression, and clinical drug trials of AD (**Table 1**).

**Table 1:** Summary description of potential retinal imaging techniques to study AD.

Techniques	Summary	Main findings	High lights	Limitation/Future Direction
**Structural imaging techniques**
Ocular fundus	Color photograph of retinal surface.	(a) Qualitative: retinopathy, optic neuropathy; (b) Retinal vascular caliber: vascular reduction, increasing variability of vessel width; (c) Global geometrical patterns: attenuation of complexity and optimality in the branching geometry; (d) Patterns of RNFL abnormalities: diffuse and wedge shape RNFL drop-out; (e) Increased drusen number, venular width gradient in peripheral retina; (f) Reduction in arterial fractal dimension in peripheral retina.	(a) Widely used; (b) Rapid; (c) Consistent; (d) Economic; (e) Non-invasive; (f) Novel fully automatically software programs: facilitating a more efficient assessment; (g) Non-mydriatic ultra-wide field retinal imaging technology: more comprehensive picture.	(a) Weak association; (b) Non-specific; (c) Longitudinal studies are needed; (d) Efficiency in measurement of retinal vasculature of ultra-wide field ocular fundus need to be established.
Optical coherence tomography (OCT)	Near-infrared light penetrates retina; interferometry resolves tissue layers.	(a) RNFL thinning; (b) Ganglion cell complex atrophy; (c) Decline in macular thickness and volume; (d) Reduction in retinal total thickness. (e) Signs of optic neuropathy: optic disk atrophy, cupping, and attenuation of neuroretinal rim.	(a) Rapid; (b) Consistent; (c) Economic; (d) Non-invasive; (e) Image registration for precise follow-up imaging; (f) Eye tracking systems to reduce motion artifacts and noise.	(a) No visualization of single cell; (b) Susceptible to ocular opacity; (c) Poor comparability between different instrument; (d) Non-specific changes to AD.
Confocal scanning laser ophthalmoscopy (cSLO)	Confocal laser beam scans of retinal surface.	(a) Significant reduction in the RNFL; (b) Reduction in neuroretinal rim; (c) Increasing vertical cup-to-disk.	(a) Non-invasive; (b) Patient-friendly (less-bright light); (c) Larger field of view; (d) Reconstruction of three-dimensional structures; (e) Increasing resolution and contrast; (f) Differentiation among neurodegenerative diseases.	(a) Poor axial resolution; (b) No visualization of single cell; (c) Expensive.
Detection of Apoptotic Retinal Cells (DARC)	Real-time visualization of apoptotic retinal cells.	Detection of increasing number of apoptotic RGCs in AD animal models.	(a) Non-invasive; (b) Quantifiable; (c) Single-cell fluorescence resolution; (d) Assessment of treatment effectiveness; (e) Screening of new drugs.	(a) Healthy dataset of apoptotic cells needs to be established; (b) Efficiency in AD patients is needed.
**Functional imaging techniques**
Pattern electroretinogram (PERG)	Using pattern-reversal stimuli and capturing retinal ganglion cell activity.	(a) Reduction in the amplitude of ERG responses; (b) Attenuation of the ERG signal.	Potential early detection of AD.	(a) Quite cumbersome; (b) Time-consuming.
Retinal oximetry	Spectrophotometric fundus imaging to measure oxygen saturation in retinal blood vessels.	Increasing arteriolar and venular oxygen saturation in MCI and moderate AD patients.	(a) Quick; (b) Safe; (c) Easily performed; (d) Patient-friendly; (e) Low variability; (f) High reproducibility.	(a) Pupil dilation is needed; (b) Sensitive to optical media opacities.
Doppler OCT	Visualization and quantification of blood flow *in vivo*.	(a) Significant reduction in the blood flow rate of the retina; (b) Narrowing retinal blood column.	(a) Rapid; (b) Consistent; (c) Economic; (d) Non-invasive;	(a) No visualization of single cell; (b) Susceptible to ocular opacity.
Retinal microperimetry	Topographic correlation between fundus details and light sensitivity of macular function.	Retinal sensitivity correlated with brain neurodegeneration among type 2 diabetes patients.	(a) Non-invasive; (b) Simple; (c) Fast; (d) Independent of the fixation and any other eye movement; (e) Independent of mood or depressive disorders; (f) High sensitivity; (g) Cost-effectiveness.	Microperimety normative database in aging healthy controls and type 2 diabetic subjects without cognitive impairment is needed.
**Animal studies**
Multiphoton microscopy	Near-infrared light emitted by fluorescent dyes of AO1-987 and CRANAD-2.	Detection of cerebral Aβ plaques in animal models.	(a) High specificity; (b) Suitability for PET scan.	(a) Highly invasive; (b) Restricted fluorescent signal.
Micron retinal imaging microscope	Visualization of fluorescence signals at high resolution and equipped with a 3-CCD camera, and specific set of filters suitable to detect curcumin fluorescence.	Dynamic pattern of plaque formation and clearance following immunotherapy.	(a) Real-time; (b) High-resolution; (c) Efficient; (d) Low-cost;	Humans studies are needed.

## Structural Imaging Methods

### Ocular Fundus or Retinal Photography

Ocular fundus or retinal photography is the classic imaging technique for investigating the retina, providing routine retinal checks in eye clinics. Ocular fundus or retinal photography is able to detect three types of retinal vascular signs: (1) qualitative retinopathy and retinal arteriolar signs, (2) changes in retinal vascular caliber and (3) changes in global geometrical patterns of the retina (Cheung et al., 2012a).

**FIGURE 1:**
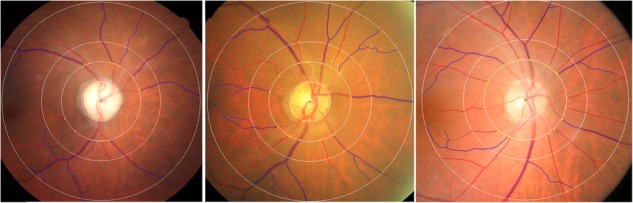
Examples of **(left)** reduced retinal vascular fractal dimension, **(middle)** attenuation retinal venular caliber and increased retinal vascular tortuosity in AD patients, **(right)** normal retinal fundus photograph. Red: arterioles; blue: venules. The measured area of retinal vascular parameters was standardized as the region from 0.5 to 2.0 disk diameters away from the disk margin Cheung et al. (2014).

Large number of studies have documented that qualitative retinal vascular signs and quantitative vascular measures, including retinopathy, vascular reduction, increasing variability of vessel width, attenuate complexity of branching characteristic, reduced optimality of the branching geometry and less tortuous venules, are associated with poorer cognitive performance based on different neuropsychological tests (Patton et al., 2007; Liew et al., 2009; Ding et al., 2011; Kim et al., 2011; Cheung et al., 2012b; Gatto et al., 2012; Ong et al., 2014; Taylor et al., 2015). The Rotterdam Study found that retinopathy was associated with AD in a cross-sectional population-based sample of individuals aged 55 years and older, whereas longitudinal data from the same study showed no association with incident AD (Schrijvers et al., 2012). Retinopathy was not significantly associated with AD when investigated in the Cardiovascular Health Study (Baker et al., 2007) and the AGES-Reykjavik Study (Qiu et al., 2010). With respect to AMD, Williams et al. (2014) showed an association between the most advanced cases and AD, whereas the association was lost after adjustment for confounding variables. Similarly, Klaver et al. (1999) found that risk of AD increased for patients with advanced stage AMD after 25.2 months follow up. However, the association was not significant following adjustment for smoking and atherosclerosis. In terms of the morphology of the ONH, a significant correlation of the optic neuropathy with the onset, severity and duration of AD was confirmed previously (Tsai et al., 1991; Hedges et al., 1996). With CRAE as an index of vessel caliber, data from the three studies (Frost et al., 2013; Cheung et al., 2014; Williams et al., 2015) were pooled and indicated a reduction in arteriolar width in AD. Increasing standard deviation of microvascular widths and microvascular attenuation in AD were found using different measures of arteriolar and venular caliber (Frost et al., 2013; Cheung et al., 2014). Three case-control studies (Frost et al., 2013; Cheung et al., 2014; Williams et al., 2015) examined the link between retinal geometric branching parameters, such as fractal dimensions, branching patterns and tortusity in AD patients. Findings from these studies reported consistent significant reduced venular, arteriolar, and total fractal dimensions, indicating a sparser network in AD (**Figure 1**). These studies have also consistently shown reduced complexity and optimality of the branching pattern. However, results of tortusity were mixed when compared AD patients with control subjects. In addition, AD patients’ fundus images illustrated different patterns of RNFL abnormalities, including diffuse and wedge shape RNFL drop-out (Lu et al., 2010).

**FIGURE 2:**
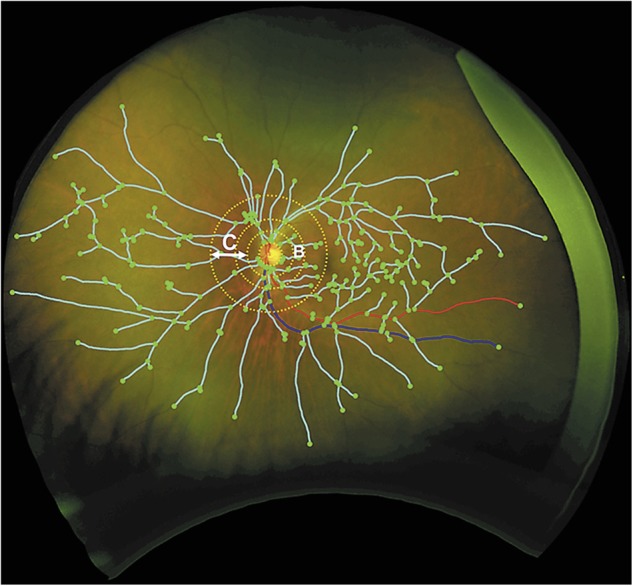
Grading of vascular parameters on ultra-wide field imaging. Dashed circles represent grading zone B: 0.5–1 OD diameter. C: 1–2 OD diameter. Turquoise lines: vascular fractal dimension; green dots: branching points; red line: arteriole; blue: venule (Csincsik et al., 2018).

Current software programs for quantitative measurement of retinal vasculature in routine retinal image are not fully automated and therefore additional efforts are needed by technicians and clinicians. Novel software programs are being developed to fully automatically measure ocular fundus features such as calibers, tortuosity and network complexity, facilitating a more efficient assessment (Cheung and Wong, 2012; Nguyen et al., 2013; Joshi et al., 2014; Cavallari et al., 2015; Abramoff et al., 2016; Walton et al., 2016). In addition, with the advent of the non-mydriatic ultra-wide field retinal imaging technology, up to 200°, rather than the common 45° or 60°, of the retina can be captured in a single shot for investigating peripheral lesions (Kernt et al., 2012), which may provide a more comprehensive picture of the overall retinal vascular structure and retina (Cheung et al., 2010). A very recent pilot study using ultra-wide field retinal imaging identified peripheral biomarkers, including markedly increased drusen number, significant increase in venular width gradient and significant decrease in arterial fractal dimension, for AD and its progression over 2 years (Csincsik et al., 2018) (**Figure 2**).

It is noteworthy that the association between retinal changes with cognitive impairment and AD is weak and non-specific (Heringa et al., 2013), partly due to the nature variation of retinal vascular parameters and common retinal lesions shared by many diseases, which may limit the usage of a single ocular fundus photography screening test for AD. However, it is possible that longitudinal analysis of retinal changes might be a potential tool to accurately detect preclinical AD or monitor AD. Further longitudinal studies are thus needed to explore this possibility and determine time course of retinal changes in AD.

### Optical Coherence Tomography (OCT)

**FIGURE 3:**
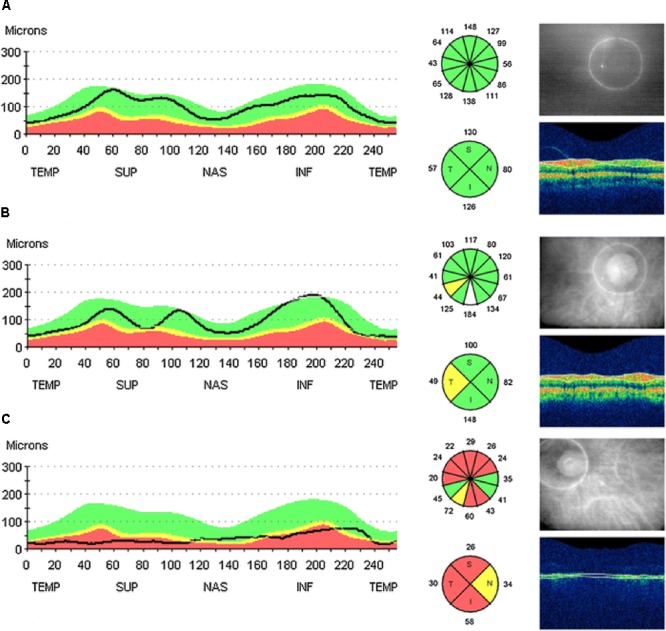
Example of peripapillary retinal nerve fiber layer (RNFL) thickness OCT measurements taken from the right eye of: a healthy control with all quadrants with **(A)** thickness normal for age, **(B)** a MCI patient with a decreased RFNL thickness in the temporal quadrant, and **(C)** a AD patient with decreased RFNL thickness in all quadrants. The line of graphic shows the RFNL thickness of the scanning circle as seen around the optic nerve head in the picture on the right. The colors represent normal distribution percentiles for age. Green: 95-5%; yellow: 5-1%; red: 1-0% (Ascaso et al., 2014).

Optical coherence tomography, a non-invasive technique providing high-resolution imaging of retina based on the principle of low coherence interferometry (Huang et al., 1991; Puliafito et al., 1995), has been extensively used to evaluate the morphological changes of the retina, such as peripapillary RNFL, macular thickness and volume, in AD. In line with the findings from post mortem studies, studies using OCT demonstrated a significant decline in peripapillary RNFL, and changes in macular thickness and volume in the eyes of patients that are progressive from MCI to AD. Results from two recent meta analysis (Coppola et al., 2015; Thomson et al., 2015) indicated that the attenuation in RNFL thickness, as observed in most studies, was significantly greater both in AD and MCI patients than that observed in the age-matched healthy controls. The mean RNFL thickness was confirmed to be reduced in AD and MCI patients in all quadrants (Parisi et al., 2001; Iseri et al., 2006; Paquet et al., 2007; Kesler et al., 2011; Kang and Kim, 2013; Kirbas et al., 2013; Ascaso et al., 2014; Polo et al., 2014; Gunes et al., 2015), but some studies reported reduction of RNFL thickness predominantly in the superior (Parisi et al., 2001; Iseri et al., 2006; Berisha et al., 2007; Kesler et al., 2011; Kang and Kim, 2013; Marziani et al., 2013; Ascaso et al., 2014; Polo et al., 2014; Gunes et al., 2015) and inferior quadrants (Parisi et al., 2001; Iseri et al., 2006; Kesler et al., 2011; Marziani et al., 2013; Ascaso et al., 2014; Polo et al., 2014; Gunes et al., 2015) (**Figure 3**). The retinal total thickness reduction in AD patients was also verified by different commercially available OCT devices (Marziani et al., 2013). Additionally to the defects in the RNFL and total retinal thickness, reduction of total macular volume was also observed in AD patients and related to the severity of the disease based on the MMSE (Iseri et al., 2006; Moreno-Ramos et al., 2013). There are, however, other studies having conflicting findings with the results described above (Paquet et al., 2007; Kesler et al., 2011; Kirbas et al., 2013; Kromer et al., 2013, 2014). With respect to the optic nerve analyzer, AD patients were more likely to show signs of optic neuropathy, such as optic disk atrophy, pathology optic disk cupping, and attenuation of the neuroretinal rim, when compared with age-matched healthy subjects (Tsai et al., 1991; Hedges et al., 1996).

More recently, OCT has evolved from older generations of TD-OCT to SD-OCT, which can capture 3D volume of retina with a higher scan speed, higher axial resolution and lower measurement variability (Leung et al., 2009). With this advance in OCT technology, precise evaluation of the sublayers of RGC is now available and moreover can be assessed using OCT automated segmentation algorithms (Schuman et al., 2009). The GC-IPL, ganglion cell layer and photoreceptor layer can be visualized using SD-OCT. This may provide additional insights into the pathology of neurodegenerative conditions. Changes in the macular GC-IPL and, macular ganglion cell complex (includes RNFL, ganglion cell layer and IPL) and photoreceptor layer are more sensitive reflections of neurodegenerative disease than the peripapillary RNFL reduction (Tan et al., 2009; Mwanza et al., 2012), since macular is the area with greatest density of cone photoreceptors cells and RGCs. Therefore, several recent studies have reported changes of specific retinal layers at the macular region in AD and supported the macular GC-IPL thinning being a new marker for detection of neurodegenerative damage in early AD and MCI (Gharbiya et al., 2014; Bayhan et al., 2015; Cheung et al., 2015; Gao et al., 2015). Two recent studies using the improved OCT indicated significant reductions in macular GCC in AD patients than the age-matched healthy controls (Marziani et al., 2013; Bayhan et al., 2015). When compared to patients with periperpillary RNFL axonal loss, patients with MCI were more specifically present with reduced macular GC-IPL (Cheung et al., 2015). The first prospective and longitudinal study found the attenuation of macular RNFL might be the earliest anatomic marker of retinal neuronal loss in the preclinical stage of AD and such loss accounted for approximate 10% of the variance in neocortical amyloidosis observed by PET imaging (Santos et al., 2018).

Limitations of these technologies should be noted when interpreting the potential utility of OCT in AD management. First, ocular opacity, such as cataract, has profound impacts on the quality of the imaging. However, newer imaging methods, such as Swept source (SS)-OCT, could overcome this limiting factor, since SS-OCT offers additional advantages over SD-OCT in penetration depth and therefore reduced sensitivity to ocular opacity (Esmaeelpour et al., 2010). Second, instrument variability and different protocols in studies may hinder the comparability between different studies. It should be noted that meta analysis of the different commercial OCT results might be offset to statistical differences in the retinal structural changes. Therefore, standardized OCT protocol to study AD is needed in the near future. Third, structural changes in retina observed in AD patients may be non-specific to AD and shared by other ocular diseases, such as decreased RNFL in glaucoma. Nevertheless, combination OCT measurements of structural changes in retina and other biomarkers could potentially be a valuable approach in AD diagnosis and prognostication.

### Confocal Scanning Laser Ophthalmoscopy (cSLO)

Confocal scanning laser ophthalmoscopy is an imaging technique and allows for reconstructing three-dimensional structures with advantages of increasing resolution and contrast. Morphological changes in the ONH measured by cSLO, including a significant reduction in the RNFL, neuroretinal rim, and increased vertical cup-to-disk, were found in AD patients in comparison to healthy subjects (Danesh-Meyer et al., 2006). In contrast to this finding, a recent study using cSLO did not document significant difference in the ONH between AD patients and healthy controls (Kurna et al., 2014). However, AD patients could be differentiated from glaucoma patients, implying the potential utility of cSLO as a new biomarker to recognize specific neurodegenerative diseases (Kurna et al., 2014). Using a lipid curcumin fluorochome and a modified point cSLO, a recent study reported the increased burden of retinal amyloid deposits in live AD patients, which might lead to a practical approach for large-scale AD screening and monitoring (Koronyo et al., 2017).

### Detection of Apoptotic Retinal Cells (DARC)

**FIGURE 4:**
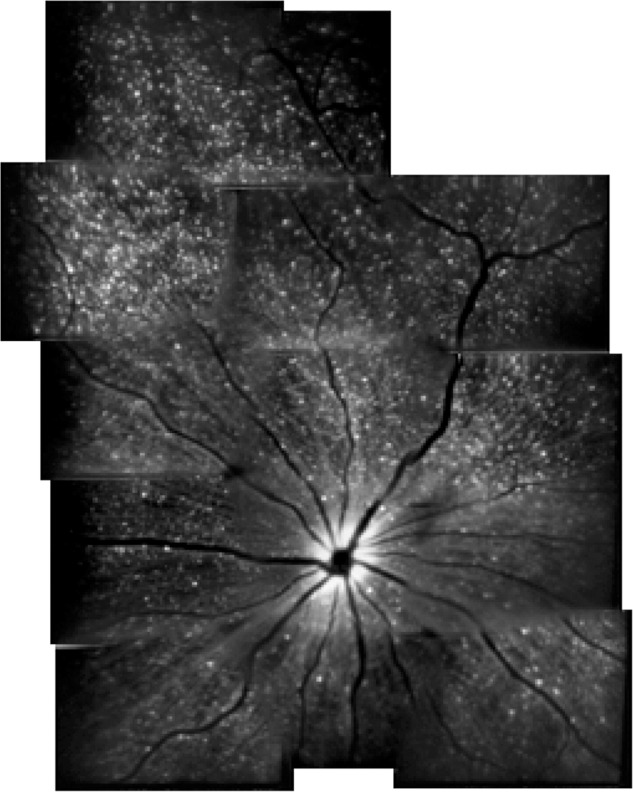
Example of detection of apoptotic retinal cells (DARC) in a rat model (Cordeiro et al., 2004).

Detection of apoptotic retinal cells is a novel imaging technology that can monitor RGC apoptosis. Although RGCs can not be real-time visualized without a marker, it has been indicated that DARC is a useful tool to detect RGC apoptosis *in vivo* in experimental animal models (Cordeiro et al., 2004, 2010; Guo et al., 2006, 2007; Maass et al., 2007) (**Figure 4**). Combined with fluorescein labeled annexin V and a cSLO, DARC allows for visualization of single RGCs undergoing apoptosis. Using DARC imaging, the number of apoptotic RGCs in a AD model significantly increased, which became even worse under oxidative stress when compared to healthy controls (Cordeiro et al., 2010). Furthermore, intraocular administration of extraneous Aβ peptides can lead to considerable RGC apoptosis *in vivo*, which can be treated and reserved by drugs targeting Aβ pathway (Guo et al., 2007). This finding promises the potential of assessing treatment effectiveness and screening new drugs by the means of DARC (Guo et al., 2006, 2007, 2014; Salt et al., 2014). Accordingly, although there is no study investigating the efficacy of DARC in patients with AD, a Phase I clinical trial of using DARC (ISRCTN59484478) in healthy and glaucoma subjects has recently been completed and DARC is believed to be used as a valuable tool in early diagnosis and treatment assessment for AD.

## Functional Imaging Methods

### Pattern Electroretinogram (PERG)

**FIGURE 5:**
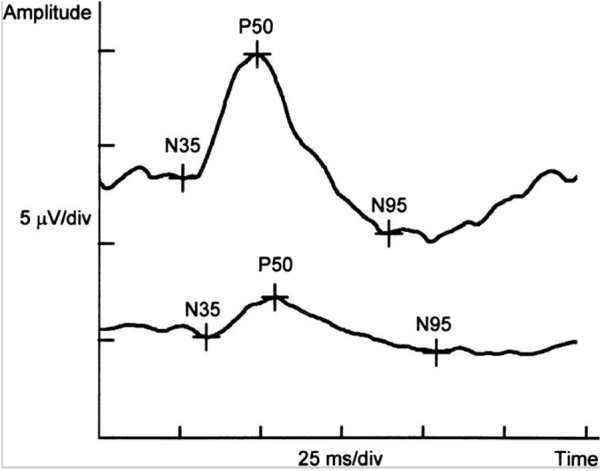
Example of PERG-P50-wave latency delay and reduced amplitudes of P50- and N95-wave in a patient with the early stage of Alzheimer’s disease (below) in comparison with the normal control (above) (Krasodomska et al., 2010).

Pattern electroretinogram allows to objectively assess ganglion cells and their axons’ electrical response to patterned stimuli (Mafei and Fiorentini, 1981; Katz et al., 1989; Trick et al., 1989). Several studies have consistently reported abnormal PERG in AD patients, such as a significant reduction in the amplitude of ERG responses and an attenuation of the ERG signal (Katz et al., 1989; Trick et al., 1989; Parisi et al., 2001), further suggesting the evidence of retinal ganglion cell dysfunction related to the structural changes in the RNFL of AD patients (Parisi et al., 2001; Parisi, 2003). However, conflicting results were also demonstrated in other studies revealing normal ERG responses in mild to moderate AD (Strenn et al., 1991; Kergoat et al., 2002). Nevertheless, PERG can be useful in early phase of the AD, when RGC dysfunction and optic nerve alteration are already existed (Krasodomska et al., 2010) (**Figure 5**). However, PERG is quite cumbersome and time-consuming, which may limit its broad availability and utility.

### Retinal Oximetry

**FIGURE 6:**
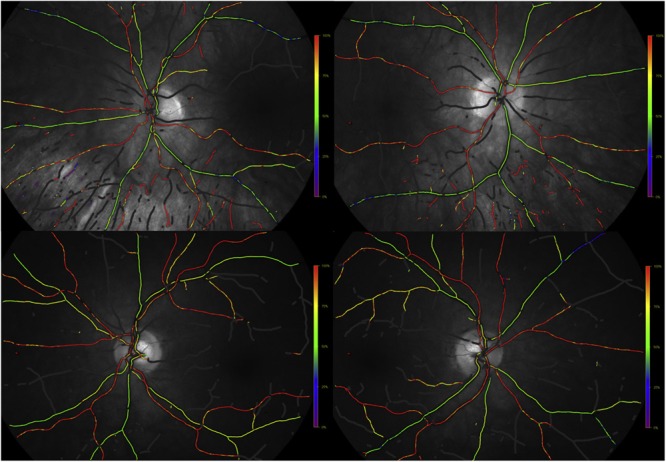
Example of fundus oximetry images from (above) a age-adjusted healthy individual and (below) an mild cognitive impairment patient (Olafsdottir et al., 2018).

Retinal oximetry is a spectrophotometric fundus imaging to measure oxygen saturation in retinal blood vessels in a non-invasive, fast and safe manner(Jani et al., 2014). Retinal oximetry can reliably and repeatedly detect changes in oxygen metabolism to reflect metabolic and functional changes in retina (Yip et al., 2014). Due to the availability of the retinal oximetry to clinical researchers in the past decade, retinal oximetry has offered new insights into several retinal diseases, such as diabetic retinopathy, glaucoma, as well as diseases of the brain, including AD (Stefansson et al., 2017). Einarsdottir et al. (2016) were the first to report oximetry abnormalities in retinas of AD patients compared with a healthy cohort. These findings have recently been confirmed and extended to patients with MCI (Olafsdottir et al., 2018) (**Figure 6**). Further studies are needed to confirm and expand the utility of retinal oximetry in AD clinical practice and trials.

### Doppler OCT

As one of the functional extensions of OCT, Doppler OCT aims to visualize and quantify blood flow *in vivo*. Significant reduction in the blood flow rate of the retina and narrowing retinal blood column had been identified through Doppler OCT in patients with AD when compared with control subjects (Berisha et al., 2007), verifying the potential association between changes of blood flow in the retina and the brain. The decreased perfusion both in the retina and the brain may affect ATP synthesis and cause oxidative stress, which resulted in neuronal death (Ruitenberg et al., 2005). Doppler OCT has been shown the potential to improve the ability to diagnose and monitor AD through ocular vascular changes.

### Retinal Microperimetry

**FIGURE 7:**
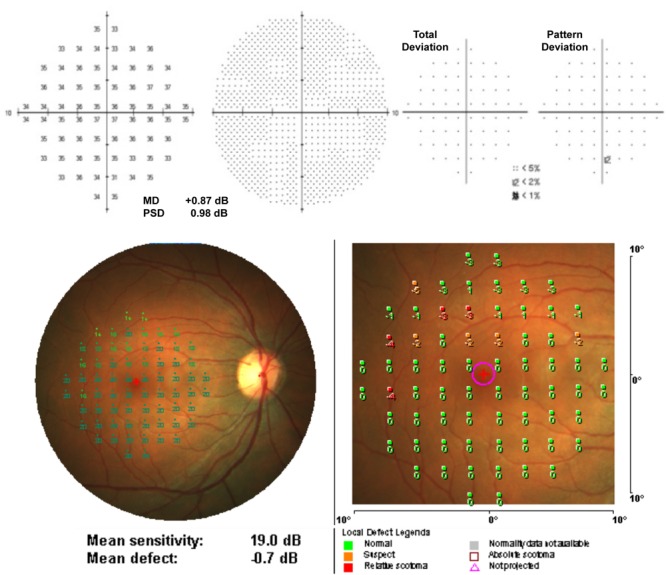
Example of a normal individual measurement from (above) Humphrey Field Analyzer (10-2 pattern, SITA Standard) and (below) MP-1 microperimetry (10-2 pattern, 4-2 strategy) (Acton et al., 2013).

Retinal microperimetry is a simple, non-invasive and fast test that measures the topographic correlation between fundus details and light sensitivity for macular function (Rohrschneider et al., 2008; Acton and Greenstein, 2013) (**Figure 7**). Independent of the fixation and any other eye movement, retinal microperimetry is a strong candidate examination for monitoring AD. It has been indicated that retinal sensitivity evaluated by retinal microperimetry correlated with brain neurodegeneration and could be a novel biomarker for predicting the risk of developing AD among type 2 diabetes patients (Ciudin et al., 2017). Some recent studies have documented more sensitivity of microperimetry in identifying early functional changes of the retina than ERG (Wu et al., 2014). Therefore, retinal microperimetry might be a cost effective way of managing the diagnosis and progression of AD in the future.

## Studies in Animal Models

Some of the promising retinal imaging technologies have been applied and tested in animal models. In a mice model expressing human mutant P301S tau, aggregation and progression of fibrillar tau in the retina were detected by cSLO over several months (Schon et al., 2012). This technique may be suitable to manage therapeutic interventions aimed at reducing fibrillar tau aggregation in the retina. Using fluorescent dyes AO1-987 and CRANAD-2 which can emit near-infrared light, multiphoton microscopy has been used to detect cerebral Aβ plaques in AD animal models (Hintersteiner et al., 2005). Despite of high specificity and suitability in PET scan (Ran et al., 2009), this technique is highly invasive and a cranial window is needed (Dong et al., 2010). Moreover, fluorescent signal is limited on the cortical surface (Meyer-Luehmann et al., 2008). Micron II retinal imaging microscope was used *in vivo* to document dynamic pattern of plaque formation and clearance following immunotherapy (Koronyo et al., 2017). This technology may be a useful tool to monitor progression and assess therapy efficiency in AD patients.

## Future Directions

Even though the current findings and applications of retinal imaging in AD patients offer a promising future, however, there are still several gaps to be filled in the future research. First, the underlying mechanisms between retinal changes and AD have not been completely elucidated, which may hinder the translation of retinal imaging into a valuable tool in AD clinical practice. Future experimental research that explores the etiology of AD and biological mechanism between retinal changes and AD is needed to facilitate the utility of retinal imaging in AD patients. Second, studies with large sample size and long-term follow-up are needed to clarify longitudinal retinal changes and its relation to AD, thus building prediction models to identify the patients at risk of developing AD. Third, a large number of novel retinal imaging technologies have been used to estimate the structure and functions of the retina, such as OCT-angiography, and retinal function imager. Very recent work has used OCT-angiography in AD patients and provided promising results that significant decreased retinal vascular density and enlarged foveal avascular zone were observed in AD group compared with the control group (Bulut et al., 2018). Further studies are needed to confirm the utility and assess the cost-effectiveness of such newer technologies in AD monitoring. Furthermore, the diagnostic and prognostic values of combining different retinal imaging technologies with neurological examinations should be investigated for the further development of multimodel for clinical use. Finally, inclusion of different types of dementia, such as Lewy body dementia, to the clinical trial is important to clarify the specific clinical implications of retinal imaging technologies in AD patients. In addition, some neurodegenerative brain disorders, such as Parkinson’s disease, have also shown morphological evidence of disruption of retinal structure and function (Archibald et al., 2009). Understanding neurodegeneration in the retina may provide not only a clearer picture of brain disorders’ onset and progression, but also promising non-invasive tools for large-scale screening and monitoring brain diseases.

## Conclusion

Structural and functional retinal changes have been suggested as the window to the neurodegenerative diseases. Retinal imaging is a non-invasive, cost-effective and highly promising method to study AD and provides new and additional important insights into AD. Future studies investigating underlying mechanisms between retinal changes and AD and the utility of combining retinal imaging technologies with neurological examinations are needed before retina imaging can be translated as a useful tool in clinical practice.

## Author Contributions

HL, ZZ, and YP conceived of the idea for the study. HL and ZZ searched and reviewed literature, drafted and revised the manuscript. YP reviewed and edited the manuscript. All authors read and approved the final manuscript.

## Conflict of Interest Statement

The authors declare that the research was conducted in the absence of any commercial or financial relationships that could be construed as a potential conflict of interest.

## Abbreviations

Aβ: amyloid-β
AβPP: amyloid-β precursor protein
AD: Alzheimer’s disease
AMD: age-related macular degeneration
BBB: blood-brain barrier
BRB: blood-retinal barrier
CFS: cerebrospinal fluid
CNS: central nervous system
CRAE: central retinal arterial equivalent
cSLO: confocal scanning laser ophthalmoscopy
DARC: detection of apoptotic retinal cells
GC-IPL: ganglion cell-inner plexiform layer
GCC: ganglion cell complex
MAP: microtubule-associated protein
MCI: mild cognitive impairment
MMSE: mini mental state examination
MRI: magnetic resonance imaging
OCT: Optical coherence tomography
ONH: optic nerve head
PERG: pattern electroretinogram
PET: positron emission tomography
PWV: pulse wave velocity
RGCs: retinal ganglion cells
RNFL: retinal nerve fiber layer
SD-OCT: spectral-domain OCT
SS-OCT: swept source OCT
TD-OCT: time-domain OCT

## References

[B1] Abramoff M. D. Lou Y. Erginay A. Clarida W. Amelon R. Folk J. C. (2016). Improved automated detection of diabetic retinopathy on a publicly available dataset through integration of deep learning. *Invest. Ophthalmol. Vis. Sci.* 57 5200–5206. 10.1167/iovs.16-19964 27701631

[B2] Acton J. H. Greenstein V. C. (2013). Fundus-driven perimetry (microperimetry) compared to conventional static automated perimetry: similarities, differences, and clinical applications. *Can. J. Ophthalmol.* 48 358–363. 10.1016/j.jcjo.2013.03.021 24093180 PMC3792399

[B3] Archibald N. K. Clarke M. P. Mosimann U. P. Burn D. J. (2009). The retina in Parkinson’s disease. *Brain* 132(Pt 5) 1128–1145. 10.1093/brain/awp068 19336464

[B4] Ascaso F. J. Cruz N. Modrego P. J. Lopez-Anton R. Santabarbara J. Pascual L. F. (2014). Retinal alterations in mild cognitive impairment and Alzheimer’s disease: an optical coherence tomography study. *J. Neurol.* 261 1522–1530. 10.1007/s00415-014-7374-z 24846203

[B5] Baker M. L. Marino Larsen E. K. Kuller L. H. Klein R. Klein B. E. Siscovick D. S. (2007). Retinal microvascular signs, cognitive function, and dementia in older persons: the cardiovascular health study. *Stroke* 38 2041–2047. 10.1161/STROKEAHA.107.483586 17525385

[B6] Bateman R. J. Xiong C. Benzinger T. L. Fagan A. M. Goate A. Fox N. C. (2012). Clinical and biomarker changes in dominantly inherited Alzheimer’s disease. *N. Engl. J. Med.* 367 795–804. 10.1056/NEJMoa1202753 22784036 PMC3474597

[B7] Bayhan H. A. Aslan Bayhan S. Celikbilek A. Tanik N. Gurdal C. (2015). Evaluation of the chorioretinal thickness changes in Alzheimer’s disease using spectral-domain optical coherence tomography. *Clin. Exp. Ophthalmol.* 43 145–151. 10.1111/ceo.12386 24995484

[B8] Beckett L. A. Harvey D. J. Gamst A. Donohue M. Kornak J. Zhang H. (2010). The Alzheimer’s disease neuroimaging initiative: annual change in biomarkers and clinical outcomes. *Alzheimers Dement.* 6 257–264. 10.1016/j.jalz.2010.03.002 20451874 PMC2867839

[B9] Berisha F. Feke G. T. Trempe C. L. McMeel J. W. Schepens C. L. (2007). Retinal abnormalities in early Alzheimer’s disease. *Invest. Ophthalmol. Vis. Sci.* 48 2285–2289. 10.1167/iovs.06-1029 17460292

[B10] Brookmeyer R. Gray S. Kawas C. (1998). Projections of Alzheimer’s disease in the United States and the public health impact of delaying disease onset. *Am. J. Public Health* 88 1337–1342. 10.2105/AJPH.88.9.1337 9736873 PMC1509089

[B11] Brookmeyer R. Johnson E. Ziegler-Graham K. Arrighi H. M. (2007). Forecasting the global burden of Alzheimer’s disease. *Alzheimers Dement.* 3 186–191. 10.1016/j.jalz.2007.04.381 19595937

[B12] Bruban J. Glotin A. L. Dinet V. Chalour N. Sennlaub F. Jonet L. (2009). Amyloid-beta(1-42) alters structure and function of retinal pigmented epithelial cells. *Aging Cell* 8 162–177. 10.1111/j.1474-9726.2009.00456.x 19239420

[B13] Bull N. D. Guidi A. Goedert M. Martin K. R. Spillantini M. G. (2012). Reduced axonal transport and increased excitotoxic retinal ganglion cell degeneration in mice transgenic for human mutant P301S tau. *PLoS One* 7:e34724. 10.1371/journal.pone.0034724 22496848 PMC3319610

[B14] Bulut M. Kurtulus F. Gozkaya O. Erol M. K. Cengiz A. Akidan M. (2018). Evaluation of optical coherence tomography angiographic findings in Alzheimer’s type dementia. *Br. J. Ophthalmol.* 102 233–237. 10.1136/bjophthalmol-2017-310476 28600299

[B15] Cai J. Qi X. Kociok N. Skosyrski S. Emilio A. Ruan Q. (2012). beta-Secretase (BACE1) inhibition causes retinal pathology by vascular dysregulation and accumulation of age pigment. *EMBO Mol. Med.* 4 980–991. 10.1002/emmm.201101084 22903875 PMC3491829

[B16] Cavallari M. Stamile C. Umeton R. Calimeri F. Orzi F. (2015). Novel method for automated analysis of retinal images: results in subjects with hypertensive retinopathy and CADASIL. *Biomed Res. Int.* 2015:752957. 10.1155/2015/752957 26167496 PMC4475739

[B17] Cheung C. Y. Ikram M. K. Sabanayagam C. Wong T. Y. (2012a). Retinal microvasculature as a model to study the manifestations of hypertension. *Hypertension* 60 1094–1103. 10.1161/HYPERTENSIONAHA.111.189142 23045470

[B18] Cheung C. Y. Ong Y. T. Hilal S. Ikram M. K. Low S. Ong Y. L. (2015). Retinal ganglion cell analysis using high-definition optical coherence tomography in patients with mild cognitive impairment and Alzheimer’s disease. *J. Alzheimers Dis.* 45 45–56. 10.3233/JAD-141659 25428254

[B19] Cheung C. Y. Ong Y. T. Ikram M. K. Ong S. Y. Li X. Hilal S. (2014). Microvascular network alterations in the retina of patients with Alzheimer’s disease. *Alzheimers Dement.* 10 135–142. 10.1016/j.jalz.2013.06.009 24439169

[B20] Cheung C. Y. Thomas G. N. Tay W. Ikram M. K. Hsu W. Lee M. L. (2012b). Retinal vascular fractal dimension and its relationship with cardiovascular and ocular risk factors. *Am. J. Ophthalmol.* 154 663.e1–674.e1. 10.1016/j.ajo.2012.04.016 22840482

[B21] Cheung N. Mosley T. Islam A. Kawasaki R. Sharrett A. R. Klein R. (2010). Retinal microvascular abnormalities and subclinical magnetic resonance imaging brain infarct: a prospective study. *Brain* 133(Pt 7) 1987–1993. 10.1093/brain/awq127 20519327 PMC2912690

[B22] Cheung N. Wong T. Y. (2012). Predicting risk of diabetic retinopathy from retinal vessel analysis: personalized medicine in transition. *Arch. Ophthalmol.* 130 783–784. 10.1001/archophthalmol.2012.727 22801841

[B23] Ciudin A. Simo-Servat O. Hernandez C. Arcos G. Diego S. Sanabria A. (2017). Retinal microperimetry: a new tool for identifying patients with type 2 diabetes at risk for developing Alzheimer disease. *Diabetes Metab. Res. Rev.* 66 3098–3104. 10.2337/db17-0382 28951388

[B24] Coppola G. Di Renzo A. Ziccardi L. Martelli F. Fadda A. Manni G. (2015). Optical coherence tomography in Alzheimer’s disease: a meta-analysis. *PLoS One* 10:e0134750. 10.1371/journal.pone.0134750 26252902 PMC4529274

[B25] Cordeiro M. F. Guo L. Coxon K. M. Duggan J. Nizari S. Normando E. M. (2010). Imaging multiple phases of neurodegeneration: a novel approach to assessing cell death *in vivo*. *Cell Death Dis.* 1:e3. 10.1038/cddis.2009.3 21364622 PMC3032512

[B26] Cordeiro M. F. Guo L. Luong V. Harding G. Wang W. Jones H. E. (2004). Real-time imaging of single nerve cell apoptosis in retinal neurodegeneration. *Proc. Natl. Acad. Sci. U.S.A.* 101 13352–13356. 10.1073/pnas.0405479101 15340151 PMC516570

[B27] Crowe M. J. Bresnahan J. C. Shuman S. L. Masters J. N. Beattie M. S. (1997). Apoptosis and delayed degeneration after spinal cord injury in rats and monkeys. *Nat. Med.* 3 73–76. 10.1038/nm0197-73 8986744

[B28] Csincsik L. MacGillivray T. J. Flynn E. Pellegrini E. Papanastasiou G. Barzegar-Befroei N. (2018). Peripheral retinal imaging biomarkers for Alzheimer’s disease: a pilot study. *Ophthalmic Res.* 59 182–192. 10.1159/000487053 29621759 PMC5985743

[B29] Danesh-Meyer H. V. Birch H. Ku J. Y. Carroll S. Gamble G. (2006). Reduction of optic nerve fibers in patients with Alzheimer disease identified by laser imaging. *Neurology* 67 1852–1854. 10.1212/01.wnl.0000244490.07925.8b 17130422

[B30] Dinet V. Bruban J. Chalour N. Maoui A. An N. Jonet L. (2012). Distinct effects of inflammation on gliosis, osmohomeostasis, and vascular integrity during amyloid beta-induced retinal degeneration. *Aging Cell* 11 683–693. 10.1111/j.1474-9726.2012.00834.x 22577879

[B31] Ding J. Strachan M. W. Fowkes F. G. Wong T. Y. Macgillivray T. J. Patton N. (2011). Association of retinal arteriolar dilatation with lower verbal memory: the Edinburgh type 2 diabetes study. *Diabetologia* 54 1653–1662. 10.1007/s00125-011-2129-1 21455727

[B32] Dong J. Revilla-Sanchez R. Moss S. Haydon P. G. (2010). Multiphoton *in vivo* imaging of amyloid in animal models of Alzheimer’s disease. *Neuropharmacology* 59 268–275. 10.1016/j.neuropharm.2010.04.007 20398680 PMC3117428

[B33] Doody R. S. Thomas R. G. Farlow M. Iwatsubo T. Vellas B. Joffe S. (2014). Phase 3 trials of solanezumab for mild-to-moderate Alzheimer’s disease. *N. Engl. J. Med.* 370 311–321. 10.1056/NEJMoa1312889 24450890

[B34] Dubois B. Feldman H. H. Jacova C. Hampel H. Molinuevo J. L. Blennow K. (2014). Advancing research diagnostic criteria for Alzheimer’s disease: the IWG-2 criteria. *Lancet Neurol.* 13 614–629. 10.1016/S1474-4422(14)70090-0 24849862

[B35] Dubois B. Hampel H. Feldman H. H. Scheltens P. Aisen P. Andrieu S. (2016). Preclinical Alzheimer’s disease: definition, natural history, and diagnostic criteria. *Alzheimers Dement.* 12 292–323. 10.1016/j.jalz.2016.02.002 27012484 PMC6417794

[B36] Einarsdottir A. B. Hardarson S. H. Kristjansdottir J. V. Bragason D. T. Snaedal J. Stefansson E. (2016). Retinal oximetry imaging in Alzheimer’s disease. *J. Alzheimers Dis.* 49 79–83. 10.3233/JAD-150457 26444785

[B37] Esmaeelpour M. Povazay B. Hermann B. Hofer B. Kajic V. Kapoor K. (2010). Three-dimensional 1060-nm OCT: choroidal thickness maps in normal subjects and improved posterior segment visualization in cataract patients. *Invest. Ophthalmol. Vis. Sci.* 51 5260–5266. 10.1167/iovs.10-5196 20445110

[B38] Faden A. I. Salzman S. (1992). Pharmacological strategies in CNS trauma. *Trends Pharmacol. Sci.* 13 29–35. 10.1016/0165-6147(92)90013-V 1311880

[B39] Fagan A. M. Xiong C. Jasielec M. S. Bateman R. J. Goate A. M. Benzinger T. L. (2014). Longitudinal change in CSF biomarkers in autosomal-dominant Alzheimer’s disease. *Sci. Transl. Med.* 6:226ra230. 10.1126/scitranslmed.3007901 24598588 PMC4038930

[B40] Feke G. T. Hyman B. T. Stern R. A. Pasquale L. R. (2015). Retinal blood flow in mild cognitive impairment and Alzheimer’s disease. *Alzheimers Dement.* 1 144–151. 10.1016/j.dadm.2015.01.004 27239502 PMC4876882

[B41] Frost S. Kanagasingam Y. Sohrabi H. Vignarajan J. Bourgeat P. Salvado O. (2013). Retinal vascular biomarkers for early detection and monitoring of Alzheimer’s disease. *Transl. Psychiatry* 3:e233. 10.1038/tp.2012.150 23443359 PMC3591002

[B42] Gao L. Liu Y. Li X. Bai Q. Liu P. (2015). Abnormal retinal nerve fiber layer thickness and macula lutea in patients with mild cognitive impairment and Alzheimer’s disease. *Arch. Gerontol. Geriatr.* 60 162–167. 10.1016/j.archger.2014.10.011 25459918

[B43] Garcia-Martin E. S. Rojas B. Ramirez A. I. de Hoz R. Salazar J. J. Yubero R. (2014). Macular thickness as a potential biomarker of mild Alzheimer’s disease. *Ophthalmology* 121 1149.e3–1151.e3. 10.1016/j.ophtha.2013.12.023 24656417

[B44] Gasparini L. Crowther R. A. Martin K. R. Berg N. Coleman M. Goedert M. (2011). Tau inclusions in retinal ganglion cells of human P301S tau transgenic mice: effects on axonal viability. *Neurobiol. Aging* 32 419–433. 10.1016/j.neurobiolaging.2009.03.002 19356824

[B45] Gatto N. M. Varma R. Torres M. Wong T. Y. Johnson P. L. Segal-Gidan F. (2012). Retinal microvascular abnormalities and cognitive function in Latino adults in Los Angeles. *Ophthalmic Epidemiol.* 19 127–136. 10.3109/09286586.2011.615452 22568425 PMC3598630

[B46] Gharbiya M. Trebbastoni A. Parisi F. Manganiello S. Cruciani F. D’Antonio F. (2014). Choroidal thinning as a new finding in Alzheimer’s disease: evidence from enhanced depth imaging spectral domain optical coherence tomography. *J. Alzheimers Dis.* 40 907–917. 10.3233/JAD-132039 24577467

[B47] Ghiso J. A. Doudevski I. Ritch R. Rostagno A. A. (2013). Alzheimer’s disease and glaucoma: mechanistic similarities and differences. *J. Glaucoma* 22(Suppl. 5) S36–S38. 10.1097/IJG.0b013e3182934af6 23733125 PMC3955061

[B48] Golzan S. M. Goozee K. Georgevsky D. Avolio A. Chatterjee P. Shen K. (2017). Retinal vascular and structural changes are associated with amyloid burden in the elderly: ophthalmic biomarkers of preclinical Alzheimer’s disease. *Alzheimers Res. Ther.* 9:13. 10.1186/s13195-017-0239-9 28253913 PMC5335799

[B49] Grundke-Iqbal I. Iqbal K. Tung Y. C. Quinlan M. Wisniewski H. M. Binder L. I. (1986). Abnormal phosphorylation of the microtubule-associated protein tau (tau) in Alzheimer cytoskeletal pathology. *Proc. Natl. Acad. Sci. U.S.A.* 83 4913–4917. 10.1073/pnas.83.13.4913 3088567 PMC323854

[B50] Gunes A. Demirci S. Tok L. Tok O. Demirci S. (2015). Evaluation of retinal nerve fiber layer thickness in Alzheimer disease using spectral-domain optical coherence tomography. *Turk. J. Med. Sci.* 45 1094–1097. 10.3906/sag-1405-114 26738353

[B51] Guo L. Davis B. Nizari S. Normando E. M. Shi H. Galvao J. (2014). Direct optic nerve sheath (DONS) application of Schwann cells prolongs retinal ganglion cell survival *in vivo*. *Cell Death Dis.* 5:e1460. 10.1038/cddis.2014.399 25321467 PMC4237238

[B52] Guo L. Duggan J. Cordeiro M. F. (2010). Alzheimer’s disease and retinal neurodegeneration. *Curr. Alzheimer Res.* 7 3–14. 10.2174/156720510790274491 20205667

[B53] Guo L. Salt T. E. Luong V. Wood N. Cheung W. Maass A. (2007). Targeting amyloid-beta in glaucoma treatment. *Proc. Natl. Acad. Sci. U.S.A.* 104 13444–13449. 10.1073/pnas.0703707104 17684098 PMC1940230

[B54] Guo L. Salt T. E. Maass A. Luong V. Moss S. E. Fitzke F. W. (2006). Assessment of neuroprotective effects of glutamate modulation on glaucoma-related retinal ganglion cell apoptosis *in vivo*. *Invest. Ophthalmol. Vis. Sci.* 47 626–633. 10.1167/iovs.05-0754 16431960 PMC2601027

[B55] Hedges T. R. III Perez Galves R. Speigelman D. Barbas N. R. Peli E. Yardley C. J. (1996). Retinal nerve fiber layer abnormalities in Alzheimer’s disease. *Acta Ophthalmol. Scand.* 74 271–275. 10.1111/j.1600-0420.1996.tb00090.x 8828725

[B56] Heringa S. M. Bouvy W. H. van den Berg E. Moll A. C. Kappelle L. J. Biessels G. J. (2013). Associations between retinal microvascular changes and dementia, cognitive functioning, and brain imaging abnormalities: a systematic review. *J. Cereb. Blood Flow Metab.* 33 983–995. 10.1038/jcbfm.2013.58 23591648 PMC3705441

[B57] Hintersteiner M. Enz A. Frey P. Jaton A. L. Kinzy W. Kneuer R. (2005). *In vivo* detection of amyloid-beta deposits by near-infrared imaging using an oxazine-derivative probe. *Nat. Biotechnol.* 23 577–583. 10.1038/nbt1085 15834405

[B58] Hinton D. R. Sadun A. A. Blanks J. C. Miller C. A. (1986). Optic-nerve degeneration in Alzheimer’s disease. *N. Engl. J. Med.* 315 485–487. 10.1056/NEJM198608213150804 3736630

[B59] Ho W. L. Leung Y. Tsang A. W. So K. F. Chiu K. Chang R. C. (2012). Review: tauopathy in the retina and optic nerve: does it shadow pathological changes in the brain? *Mol. Vis.* 18 2700–2710. 23170062 PMC3501278

[B60] Holtzman D. M. Morris J. C. Goate A. M. (2011). Alzheimer’s disease: the challenge of the second century. *Sci. Transl. Med.* 3:77sr1. 10.1126/scitranslmed.3002369 21471435 PMC3130546

[B61] Huang D. Swanson E. A. Lin C. P. Schuman J. S. Stinson W. G. Chang W. (1991). Optical coherence tomography. *Science* 254 1178–1181. 10.1126/science.1957169 1957169 PMC4638169

[B62] Iseri P. K. Altinas O. Tokay T. Yuksel N. (2006). Relationship between cognitive impairment and retinal morphological and visual functional abnormalities in Alzheimer disease. *J. Neuroophthalmol.* 26 18–24. 10.1097/01.wno.0000204645.56873.26 16518161

[B63] Jahn T. R. Makin O. S. Morris K. L. Marshall K. E. Tian P. Sikorski P. (2010). The common architecture of cross-beta amyloid. *J. Mol. Biol.* 395 717–727. 10.1016/j.jmb.2009.09.039 19781557

[B64] James O. G. Doraiswamy P. M. Borges-Neto S. (2015). PET imaging of tau pathology in Alzheimer’s disease and tauopathies. *Front. Neurol.* 6:38 10.3389/fneur.2015.00038 PMC4353301 25806018

[B65] Jani P. D. Mwanza J. C. Billow K. B. Waters A. M. Moyer S. Garg S. (2014). Normative values and predictors of retinal oxygen saturation. *Retina* 34 394–401. 10.1097/IAE.0b013e3182979e7b 23842102

[B66] Jellinger K. A. Attems J. (2015). Challenges of multimorbidity of the aging brain: a critical update. *J. Neural Transm.* 122 505–521. 10.1007/s00702-014-1288-x 25091618

[B67] Joshi V. Agurto C. VanNess R. Nemeth S. Soliz P. Barriga S. (2014). Comprehensive automatic assessment of retinal vascular abnormalities for computer-assisted retinopathy grading. *Conf. Proc. IEEE Eng. Med. Biol. Soc.* 2014 6320–6323. 10.1109/EMBC.2014.6945074 25571442

[B68] Kang B. H. Kim J. I. (2013). Decreased retinal thickness in patients with Alzheimer’s disease. *J. Korean Neurol. Assoc.* 31 173–177.

[B69] Katz B. Rimmer S. Iragui V. Katzman R. (1989). Abnormal pattern electroretinogram in Alzheimer’s disease: evidence for retinal ganglion cell degeneration? *Ann. Neurol.* 26 221–225. 10.1002/ana.410260207 2774509

[B70] Kaur C. Foulds W. S. Ling E. A. (2008). Blood-retinal barrier in hypoxic ischaemic conditions: basic concepts, clinical features and management. *Prog. Retin. Eye Res.* 27 622–647. 10.1016/j.preteyeres.2008.09.003 18940262

[B71] Kergoat H. Kergoat M. J. Justino L. Chertkow H. Robillard A. Bergman H. (2002). Visual retinocortical function in dementia of the Alzheimer type. *Gerontology* 48 197–203. 10.1159/000058350 12053107

[B72] Kernt M. Hadi I. Pinter F. Seidensticker F. Hirneiss C. Haritoglou C. (2012). Assessment of diabetic retinopathy using nonmydriatic ultra-widefield scanning laser ophthalmoscopy (Optomap) compared with ETDRS 7-field stereo photography. *Diabetes Care* 35 2459–2463. 10.2337/dc12-0346 22912430 PMC3507573

[B73] Kesler A. Vakhapova V. Korczyn A. D. Naftaliev E. Neudorfer M. (2011). Retinal thickness in patients with mild cognitive impairment and Alzheimer’s disease. *Clin. Neurol. Neurosurg.* 113 523–526. 10.1016/j.clineuro.2011.02.014 21454010

[B74] Kim D. H. Newman A. B. Hajjar I. Strotmeyer E. S. Klein R. Newton E. (2011). Retinal microvascular signs and functional loss in older persons: the cardiovascular health study. *Stroke* 42 1589–1595. 10.1161/STROKEAHA.110.605261 21493913 PMC3127407

[B75] Kirbas S. Turkyilmaz K. Anlar O. Tufekci A. Durmus M. (2013). Retinal nerve fiber layer thickness in patients with Alzheimer disease. *J. Neuroophthalmol.* 33 58–61. 10.1097/WNO.0b013e318267fd5f 22918296

[B76] Klaver C. C. Ott A. Hofman A. Assink J. J. Breteler M. M. de Jong P. T. (1999). Is age-related maculopathy associated with Alzheimer’s Disease? The Rotterdam study. *Am. J. Epidemiol.* 150 963–968. 10.1093/oxfordjournals.aje.a010105 10547142

[B77] Koronyo Y. Biggs D. Barron E. Boyer D. S. Pearlman J. A. Au W. J. (2017). Retinal amyloid pathology and proof-of-concept imaging trial in Alzheimer’s disease. *JCI Insight* 2:93621. 10.1172/jci.insight.93621 28814675 PMC5621887

[B78] Koronyo Y. Salumbides B. C. Black K. L. Koronyo-Hamaoui M. (2012). Alzheimer’s disease in the retina: imaging retinal abeta plaques for early diagnosis and therapy assessment. *Neurodegener. Dis.* 10 285–293. 10.1159/000335154 22343730

[B79] Koronyo-Hamaoui M. Koronyo Y. Ljubimov A. V. Miller C. A. Ko M. K. Black K. L. (2011). Identification of amyloid plaques in retinas from Alzheimer’s patients and noninvasive *in vivo* optical imaging of retinal plaques in a mouse model. *Neuroimage* 54(Suppl. 1) S204–S217. 10.1016/j.neuroimage.2010.06.020 20550967 PMC2991559

[B80] Krasodomska K. Lubinski W. Potemkowski A. Honczarenko K. (2010). Pattern electroretinogram (PERG) and pattern visual evoked potential (PVEP) in the early stages of Alzheimer’s disease. *Doc. Ophthalmol.* 121 111–121. 10.1007/s10633-010-9238-x 20549299 PMC2941083

[B81] Kromer R. Serbecic N. Hausner L. Froelich L. Aboul-Enein F. Beutelspacher S. C. (2014). Detection of retinal nerve fiber layer defects in Alzheimer’s disease using SD-OCT. *Front. Psychiatry* 5:22. 10.3389/fpsyt.2014.00022 24616709 PMC3934110

[B82] Kromer R. Serbecic N. Hausner L. Froelich L. Beutelspacher S. C. (2013). Comparison of visual evoked potentials and retinal nerve fiber layer thickness in Alzheimer’s disease. *Front. Neurol.* 4:203 10.3389/fneur.2013.00203 PMC3864196 24379800

[B83] Kurna S. A. Akar G. Altun A. Agirman Y. Gozke E. Sengor T. (2014). Confocal scanning laser tomography of the optic nerve head on the patients with Alzheimer’s disease compared to glaucoma and control. *Int. Ophthalmol.* 34 1203–1211. 10.1007/s10792-014-0004-z 25284015

[B84] Leung C. K. Cheung C. Y. Weinreb R. N. Qiu Q. Liu S. Li H. (2009). Retinal nerve fiber layer imaging with spectral-domain optical coherence tomography: a variability and diagnostic performance study. *Ophthalmology* 116 1257–1263 1263.e1–1263.e2. 10.1016/j.ophtha.2009.04.013 19464061

[B85] Levkovitch-Verbin H. Quigley H. A. Kerrigan-Baumrind L. A. D’Anna S. A. Kerrigan D. Pease M. E. (2001). Optic nerve transection in monkeys may result in secondary degeneration of retinal ganglion cells. *Invest. Ophthalmol. Vis. Sci.* 42 975–982. 11274074

[B86] Levkovitch-Verbin H. Quigley H. A. Martin K. R. Zack D. J. Pease M. E. Valenta D. F. (2003). A model to study differences between primary and secondary degeneration of retinal ganglion cells in rats by partial optic nerve transection. *Invest. Ophthalmol. Vis. Sci.* 44 3388–3393. 10.1167/iovs.02-0646 12882786

[B87] Li L. Luo J. Chen D. Tong J. B. Zeng L. P. Cao Y. Q. (2016). BACE1 in the retina: a sensitive biomarker for monitoring early pathological changes in Alzheimer’s disease. *Neural Regen. Res.* 11 447–453. 10.4103/1673-5374.179057 27127484 PMC4829010

[B88] Liew G. Mitchell P. Wong T. Y. Lindley R. I. Cheung N. Kaushik S. (2009). Retinal microvascular signs and cognitive impairment. *J. Am. Geriatr. Soc.* 57 1892–1896. 10.1111/j.1532-5415.2009.02459.x 19737331

[B89] Liu B. Rasool S. Yang Z. Glabe C. G. Schreiber S. S. Ge J. (2009). Amyloid-peptide vaccinations reduce {beta}-amyloid plaques but exacerbate vascular deposition and inflammation in the retina of Alzheimer’s transgenic mice. *Am. J. Pathol.* 175 2099–2110. 10.2353/ajpath.2009.090159 19834067 PMC2774073

[B90] London A. Benhar I. Schwartz M. (2013). The retina as a window to the brain-from eye research to CNS disorders. *Nat. Rev. Neurol.* 9 44–53. 10.1038/nrneurol.2012.227 23165340

[B91] Lu Y. Li Z. Zhang X. Ming B. Jia J. Wang R. (2010). Retinal nerve fiber layer structure abnormalities in early Alzheimer’s disease: evidence in optical coherence tomography. *Neurosci. Lett.* 480 69–72. 10.1016/j.neulet.2010.06.006 20609426

[B92] Maass A. von Leithner P. L. Luong V. Guo L. Salt T. E. Fitzke F. W. (2007). Assessment of rat and mouse RGC apoptosis imaging *in vivo* with different scanning laser ophthalmoscopes. *Curr. Eye Res.* 32 851–861. 10.1080/02713680701585872 17963105 PMC2601026

[B93] MacCormick I. J. Czanner G. Faragher B. (2015). Developing retinal biomarkers of neurological disease: an analytical perspective. *Biomark. Med.* 9 691–701. 10.2217/bmm.15.17 26174843 PMC4822679

[B94] Mafei L. Fiorentini A. (1981). Electroretinographic responses to alternating gratings before and after section of the optic nerve. *Science* 211 953–955. 10.1126/science.7466369 7466369

[B95] Marziani E. Pomati S. Ramolfo P. Cigada M. Giani A. Mariani C. (2013). Evaluation of retinal nerve fiber layer and ganglion cell layer thickness in Alzheimer’s disease using spectral-domain optical coherence tomography. *Invest. Ophthalmol. Vis. Sci* 54 5953–5958. 10.1167/iovs.13-12046 23920375

[B96] Masters C. L. Simms G. Weinman N. A. Multhaup G. McDonald B. L. Beyreuther K. (1985). Amyloid plaque core protein in Alzheimer disease and Down syndrome. *Proc. Natl. Acad. Sci. U.S.A.* 82 4245–4249. 10.1073/pnas.82.12.4245 3159021 PMC397973

[B97] McKhann G. Drachman D. Folstein M. Katzman R. Price D. Stadlan E. M. (1984). Clinical diagnosis of Alzheimer’s disease: report of the NINCDS-ADRDA work group under the auspices of department of health and human services task force on Alzheimer’s disease. *Neurology* 34 939–944. 10.1212/WNL.34.7.939 6610841

[B98] Meyer-Luehmann M. Spires-Jones T. L. Prada C. Garcia-Alloza M. de Calignon A. Rozkalne A. (2008). Rapid appearance and local toxicity of amyloid-beta plaques in a mouse model of Alzheimer’s disease. *Nature* 451 720–724. 10.1038/nature06616 18256671 PMC3264491

[B99] Moore K. M. Girens R. E. Larson S. K. Jones M. R. Restivo J. L. Holtzman D. M. (2016). A spectrum of exercise training reduces soluble Abeta in a dose-dependent manner in a mouse model of Alzheimer’s disease. *Neurobiol. Dis.* 85 218–224. 10.1016/j.nbd.2015.11.004 26563933

[B100] Moreno-Ramos T. Benito-Leon J. Villarejo A. Bermejo-Pareja F. (2013). Retinal nerve fiber layer thinning in dementia associated with Parkinson’s disease, dementia with Lewy bodies, and Alzheimer’s disease. *J. Alzheimers Dis.* 34 659–664. 10.3233/JAD-121975 23271313

[B101] Morin P. J. Abraham C. R. Amaratunga A. Johnson R. J. Huber G. Sandell J. H. (1993). Amyloid precursor protein is synthesized by retinal ganglion cells, rapidly transported to the optic nerve plasma membrane and nerve terminals, and metabolized. *J. Neurochem.* 61 464–473. 10.1111/j.1471-4159.1993.tb02147.x 7687653

[B102] Mwanza J. C. Durbin M. K. Budenz D. L. Sayyad F. E. Chang R. T. Neelakantan A. (2012). Glaucoma diagnostic accuracy of ganglion cell-inner plexiform layer thickness: comparison with nerve fiber layer and optic nerve head. *Ophthalmology* 119 1151–1158. 10.1016/j.ophtha.2011.12.014 22365056

[B103] Nguyen U. T. Bhuiyan A. Park L. A. Kawasaki R. Wong T. Y. Wang J. J. (2013). Automated quantification of retinal arteriovenous nicking from colour fundus images. *Conf. Proc. IEEE Eng. Med. Biol. Soc.* 2013 5865–5868. 10.1109/EMBC.2013.6610886 24111073

[B104] Ning A. Cui J. To E. Ashe K. H. Matsubara J. (2008). Amyloid-beta deposits lead to retinal degeneration in a mouse model of Alzheimer disease. *Invest. Ophthalmol. Vis. Sci.* 49 5136–5143. 10.1167/iovs.08-1849 18566467 PMC3947384

[B105] Olafsdottir O. B. Saevarsdottir H. S. Hardarson S. H. Hannesdottir K. H. Traustadottir V. D. Karlsson R. A. (2018). Retinal oxygen metabolism in patients with mild cognitive impairment. *Alzheimers Dement.* 10 340–345. 10.1016/j.dadm.2018.03.002 PMC6024244 30014033

[B106] Ong Y. T. Hilal S. Cheung C. Y. Xu X. Chen C. Venketasubramanian N. (2014). Retinal vascular fractals and cognitive impairment. *Dement. Geriatr. Cogn. Dis. Extra* 4 305–313. 10.1159/000363286 25298774 PMC4176466

[B107] Paquet C. Boissonnot M. Roger F. Dighiero P. Gil R. Hugon J. (2007). Abnormal retinal thickness in patients with mild cognitive impairment and Alzheimer’s disease. *Neurosci. Lett.* 420 97–99. 10.1016/j.neulet.2007.02.090 17543991

[B108] Parisi V. (2003). Correlation between morphological and functional retinal impairment in patients affected by ocular hypertension, glaucoma, demyelinating optic neuritis and Alzheimer’s disease. *Semin. Ophthalmol.* 18 50–57. 14566623 10.1076/soph.18.2.50.15855

[B109] Parisi V. Restuccia R. Fattapposta F. Mina C. Bucci M. G. Pierelli F. (2001). Morphological and functional retinal impairment in Alzheimer’s disease patients. *Clin. Neurophysiol.* 112 1860–1867. 10.1016/S1388-2457(01)00620-4 11595144

[B110] Park S. W. Kim J. H. Mook-Jung I. Kim K. W. Park W. J. Park K. H. (2014). Intracellular amyloid beta alters the tight junction of retinal pigment epithelium in 5XFAD mice. *Neurobiol. Aging* 35 2013–2020. 10.1016/j.neurobiolaging.2014.03.008 24709310

[B111] Parnell M. Guo L. Abdi M. Cordeiro M. F. (2012). Ocular manifestations of Alzheimer’s disease in animal models. *Int. J. Alzheimers Dis.* 2012:786494. 10.1155/2012/786494 22666623 PMC3362039

[B112] Pase M. P. Herbert A. Grima N. A. Pipingas A. O’Rourke M. F. (2012). Arterial stiffness as a cause of cognitive decline and dementia: a systematic review and meta-analysis. *Intern. Med. J.* 42 808–815. 10.1111/j.1445-5994.2011.02645.x 22151013

[B113] Patton N. Pattie A. MacGillivray T. Aslam T. Dhillon B. Gow A. (2007). The association between retinal vascular network geometry and cognitive ability in an elderly population. *Invest. Ophthalmol. Vis. Sci.* 48 1995–2000. 10.1167/iovs.06-1123 17460252

[B114] Perez S. E. Lumayag S. Kovacs B. Mufson E. J. Xu S. (2009). Beta-amyloid deposition and functional impairment in the retina of the APPswe/PS1DeltaE9 transgenic mouse model of Alzheimer’s disease. *Invest. Ophthalmol. Vis. Sci.* 50 793–800. 10.1167/iovs.08-2384 18791173 PMC3697019

[B115] Polanco J. C. Li C. Bodea L. G. Martinez-Marmol R. Meunier F. A. Gotz J. (2018). Amyloid-beta and tau complexity - towards improved biomarkers and targeted therapies. *Nat. Rev. Neurol.* 14 22–39. 10.1038/nrneurol.2017.162 29242522

[B116] Polo V. Garcia-Martin E. Bambo M. P. Pinilla J. Larrosa J. M. Satue M. (2014). Reliability and validity of Cirrus and Spectralis optical coherence tomography for detecting retinal atrophy in Alzheimer’s disease. *Eye* 28 680–690. 10.1038/eye.2014.51 24625377 PMC4058616

[B117] Prince M. Wimo A. Guerchet M. Claire A. YuTzu W. Matthew P. (2015). *World Alzheimer Report 2015: The Global Impact of Dementia: An Analysis of Prevalence, Incidence, Cost and Trends*. London: Alzheimer’s Disease International.

[B118] Puliafito C. A. Hee M. R. Lin C. P. Reichel E. Schuman J. S. Duker J. S. (1995). Imaging of macular diseases with optical coherence tomography. *Ophthalmology* 102 217–229. 10.1016/S0161-6420(95)31032-9 7862410

[B119] Qiu C. Cotch M. F. Sigurdsson S. Jonsson P. V. Jonsdottir M. K. Sveinbjrnsdottir S. (2010). Cerebral microbleeds, retinopathy, and dementia: the AGES-Reykjavik study. *Neurology* 75 2221–2228. 10.1212/WNL.0b013e3182020349 21172845 PMC3013588

[B120] Ran C. Xu X. Raymond S. B. Ferrara B. J. Neal K. Bacskai B. J. (2009). Design, synthesis, and testing of difluoroboron-derivatized curcumins as near-infrared probes for *in vivo* detection of amyloid-beta deposits. *J. Am. Chem. Soc.* 131 15257–15261. 10.1021/ja9047043 19807070 PMC2784241

[B121] Rohrschneider K. Bultmann S. Springer C. (2008). Use of fundus perimetry (microperimetry) to quantify macular sensitivity. *Prog. Retin. Eye Res.* 27 536–548. 10.1016/j.preteyeres.2008.07.003 18723109

[B122] Ruitenberg A. den Heijer T. Bakker S. L. van Swieten J. C. Koudstaal P. J. Hofman A. (2005). Cerebral hypoperfusion and clinical onset of dementia: the Rotterdam study. *Ann. Neurol.* 57 789–794. 10.1002/ana.20493 15929050

[B123] Salloway S. Sperling R. Fox N. C. Blennow K. Klunk W. Raskind M. (2014). Two phase 3 trials of bapineuzumab in mild-to-moderate Alzheimer’s disease. *N. Engl. J. Med.* 370 322–333. 10.1056/NEJMoa1304839 24450891 PMC4159618

[B124] Salobrar-Garcia E. Hoyas I. Leal M. de Hoz R. Rojas B. Ramirez A. I. (2015). Analysis of retinal peripapillary segmentation in early Alzheimer’s disease patients. *Biomed Res. Int.* 2015:636548. 10.1155/2015/636548 26557684 PMC4628738

[B125] Salt T. E. Nizari S. Cordeiro M. F. Russ H. Danysz W. (2014). Effect of the Abeta aggregation modulator MRZ-99030 on retinal damage in an animal model of glaucoma. *Neurotox. Res.* 26 440–446. 10.1007/s12640-014-9488-6 25106883

[B126] Santos C. Y. Johnson L. N. Sinoff S. E. Festa E. K. Heindel W. C. Snyder P. J. (2018). Change in retinal structural anatomy during the preclinical stage of Alzheimer’s disease. *Alzheimers Dement.* 10 196–209. 10.1016/j.dadm.2018.01.003 29780864 PMC5956814

[B127] Schon C. Hoffmann N. A. Ochs S. M. Burgold S. Filser S. Steinbach S. (2012). Long-term *in vivo* imaging of fibrillar tau in the retina of P301S transgenic mice. *PLoS One* 7:e53547. 10.1371/journal.pone.0053547 23300938 PMC3534024

[B128] Schrijvers E. M. Buitendijk G. H. Ikram M. K. Koudstaal P. J. Hofman A. Vingerling J. R. (2012). Retinopathy and risk of dementia: the Rotterdam study. *Neurology* 79 365–370. 10.1212/WNL.0b013e318260cd7e 22786586 PMC3400091

[B129] Schuman S. G. Koreishi A. F. Farsiu S. Jung S. H. Izatt J. A. Toth C. A. (2009). Photoreceptor layer thinning over drusen in eyes with age-related macular degeneration imaged *in vivo* with spectral-domain optical coherence tomography. *Ophthalmology* 116 488.e–496.e. 10.1016/j.ophtha.2008.10.006 19167082 PMC2695995

[B130] Schwartz M. Belkin M. Yoles E. Solomon A. (1996). Potential treatment modalities for glaucomatous neuropathy: neuroprotection and neuroregeneration. *J. Glaucoma* 5 427–432. 10.1097/00061198-199612000-00012 8946301

[B131] Sevigny J. Chiao P. Bussiere T. Weinreb P. H. Williams L. Maier M. (2016). The antibody aducanumab reduces Abeta plaques in Alzheimer’s disease. *Nature* 537 50–56. 10.1038/nature19323 27582220

[B132] Siemers E. R. Sundell K. L. Carlson C. Case M. Sethuraman G. Liu-Seifert H. (2016). Phase 3 solanezumab trials: secondary outcomes in mild Alzheimer’s disease patients. *Alzheimers Dement.* 12 110–120. 10.1016/j.jalz.2015.06.1893 26238576

[B133] Sperling R. A. Aisen P. S. Beckett L. A. Bennett D. A. Craft S. Fagan A. M. (2011a). Toward defining the preclinical stages of Alzheimer’s disease: recommendations from the National Institute on Aging-Alzheimer’s Association workgroups on diagnostic guidelines for Alzheimer’s disease. *Alzheimers Dement.* 7 280–292. 10.1016/j.jalz.2011.03.003 21514248 PMC3220946

[B134] Sperling R. A. Jack C. R. Jr. Aisen P. S. (2011b). Testing the right target and right drug at the right stage. *Sci. Transl. Med.* 3:111cm133. 10.1126/scitranslmed.3002609 22133718 PMC3752906

[B135] Stefansson E. Olafsdottir O. B. Einarsdottir A. B. Eliasdottir T. S. Eysteinsson T. Vehmeijer W. (2017). Retinal oximetry discovers novel biomarkers in retinal and brain diseases. *Invest. Ophthalmol. Vis. Sci.* 58 BIO227–BIO233. 10.1167/iovs.17-21776 28810002

[B136] Strenn K. Dal-Bianco P. Weghaupt H. Koch G. Vass C. Gottlob I. (1991). Pattern electroretinogram and luminance electroretinogram in Alzheimer’s disease. *J. Neural Transm. Suppl.* 33 73–80. 10.1007/978-3-7091-9135-4_12 1753255

[B137] Tan O. Chopra V. Lu A. T. Schuman J. S. Ishikawa H. Wollstein G. (2009). Detection of macular ganglion cell loss in glaucoma by Fourier-domain optical coherence tomography. *Ophthalmology* 116 2305.e2–2314.e2. 10.1016/j.ophtha.2009.05.025 19744726 PMC2787911

[B138] Taylor A. M. MacGillivray T. J. Henderson R. D. Ilzina L. Dhillon B. Starr J. M. (2015). Retinal vascular fractal dimension, childhood IQ, and cognitive ability in old age: the Lothian Birth Cohort Study 1936. *PLoS One* 10:e0121119. 10.1371/journal.pone.0121119 25816017 PMC4376388

[B139] Terry R. D. DeTeresa R. Hansen L. A. (1987). Neocortical cell counts in normal human adult aging. *Ann. Neurol.* 21 530–539. 10.1002/ana.410210603 3606042

[B140] Thomson K. L. Yeo J. M. Waddell B. Cameron J. R. Pal S. (2015). A systematic review and meta-analysis of retinal nerve fiber layer change in dementia, using optical coherence tomography. *Alzheimers Dement.* 1 136–143. 10.1016/j.dadm.2015.03.001 27239501 PMC4876885

[B141] Trick G. L. Barris M. C. Bickler-Bluth M. (1989). Abnormal pattern electroretinograms in patients with senile dementia of the Alzheimer type. *Ann. Neurol.* 26 226–231. 10.1002/ana.410260208 2774510

[B142] Tsai C. S. Ritch R. Schwartz B. Lee S. S. Miller N. R. Chi T. (1991). Optic nerve head and nerve fiber layer in Alzheimer’s disease. *Arch. Ophthalmol.* 109 199–204. 10.1001/archopht.1991.01080020045040 1993028

[B143] Vecino E. Rodriguez F. D. Ruzafa N. Pereiro X. Sharma S. C. (2016). Glia-neuron interactions in the mammalian retina. *Prog. Retin. Eye Res.* 51 1–40. 10.1016/j.preteyeres.2015.06.003 26113209

[B144] Walton O. B. Garoon R. B. Weng C. Y. Gross J. Young A. K. Camero K. A. (2016). Evaluation of automated teleretinal screening program for diabetic retinopathy. *JAMA Ophthalmol.* 134 204–209. 10.1001/jamaophthalmol.2015.5083 26720694

[B145] Wang J. Ohno-Matsui K. Yoshida T. Shimada N. Ichinose S. Sato T. (2009). Amyloid-beta up-regulates complement factor B in retinal pigment epithelial cells through cytokines released from recruited macrophages/microglia: another mechanism of complement activation in age-related macular degeneration. *J. Cell. Physiol.* 220 119–128. 10.1002/jcp.21742 19277984

[B146] Williams M. A. McGowan A. J. Cardwell C. R. Cheung C. Y. Craig D. Passmore P. (2015). Retinal microvascular network attenuation in Alzheimer’s disease. *Alzheimers Dement.* 1 229–235. 10.1016/j.dadm.2015.04.001 26634224 PMC4629099

[B147] Williams M. A. Silvestri V. Craig D. Passmore A. P. Silvestri G. (2014). The prevalence of age-related macular degeneration in Alzheimer’s disease. *J. Alzheimers Dis.* 42 909–914. 10.3233/JAD-140243 25024309

[B148] Wu Z. Ayton L. N. Guymer R. H. Luu C. D. (2014). Comparison between multifocal electroretinography and microperimetry in age-related macular degeneration. *Invest. Ophthalmol. Vis. Sci.* 55 6431–6439. 10.1167/iovs.14-14407 25159206

[B149] Yip W. Siantar R. Perera S. A. Milastuti N. Ho K. K. Tan B. (2014). Reliability and determinants of retinal vessel oximetry measurements in healthy eyes. *Invest. Ophthalmol. Vis. Sci.* 55 7104–7110. 10.1167/iovs.13-13854 25301879

[B150] Yoles E. Schwartz M. (1998). Degeneration of spared axons following partial white matter lesion: implications for optic nerve neuropathies. *Exp. Neurol.* 153 1–7. 10.1006/exnr.1998.6811 9743562

[B151] Zhang-Nunes S. X. Maat-Schieman M. L. van Duinen S. G. Roos R. A. Frosch M. P. Greenberg S. M. (2006). The cerebral beta-amyloid angiopathies: hereditary and sporadic. *Brain Pathol.* 16 30–39. 10.1111/j.1750-3639.2006.tb00559.x 16612980 PMC8095991

[B152] Zlokovic B. V. (2011). Neurovascular pathways to neurodegeneration in Alzheimer’s disease and other disorders. *Nat. Rev. Neurosci.* 12 723–738. 10.1038/nrn3114 22048062 PMC4036520

